# Cervical cancer patients presentation and survival in the only oncology referral hospital, Ethiopia: a retrospective cohort study

**DOI:** 10.1186/s13027-017-0171-4

**Published:** 2017-11-29

**Authors:** Muluken Gizaw, Adamu Addissie, Sefonias Getachew, Wondimu Ayele, Israel Mitiku, Ulrike Moelle, Tigist Yusuf, Mathias Begoihn, Mathewos Assefa, Ahmedin Jemal, Eva Johanna Kantelhardt

**Affiliations:** 1 Institute of Medical Epidemiology, Biostatistics and Informatics, Martin-Luther-University, Halle (Saale), Germany; 2 Department of Preventive Medicine, School of Public Health, Addis Ababa University, Addis Ababa, Ethiopia; 3 Department of Intramural Research, American Cancer Society, Atlanta, GA USA; 4 Department of Public Health, College of Medicine and Health Sciences, Wollo University, Dessie, Ethiopia; 5 Department of Gynecology, Martin-Luther-University, Halle (Saale), Germany; 6 Radiotherapy Center, School of Medicine, Addis Ababa University, Addis Ababa, Ethiopia

**Keywords:** Uterine cervical neoplasms, HIV, Survival, Africa, Ethiopia

## Abstract

**Background:**

Women infected with Human Immune Deficiency Virus (HIV) are assumed to be at higher risk of developing Cervical Cancer (CC). This is due to a rapid progression of pre-invasive to invasive lesions. However, evidences suggest, due to the availability of antiretroviral therapy (ART) and care services; an improved survival and treatment outcome of CC patients (CCPs) with HIV infection is expected.

**Objective:**

The aim of this study is to examine the clinical characteristics and survival of of CCPs registered at the radiotherapy center of Tikur Anbessa Specialized Hospital (TASH), Addis Ababa University, Ethiopia.

**Methods:**

We conducted a retrospective cohort study. Data from 1655 CCPs diagnosed between September 2008 and September 2012 were included. The primary endpoint was death from any cause. Kaplan-Meier estimates were compared using the log-rank test. Cox proportional hazards regression model was used to identify predictors of death. Data were analyzed using STATA version IC/14.

**Results:**

The mean age of all patients was 49 years (SD = 11.6 years). Of all CCPs, 139 (8.4%) were HIV positive, 372 (22.5%) patients had a known negative HIV status and 1144 (69.1%) patients were asymptomatic with unknown HIV status. Due to late stage and waiting times, only 13.5% of the patients received curative radiotherapy doses. HIV-positive CCPs presented more often with advanced disease compared to HIV negative CCPs ((44.6%) versus 39.7%, *p* = 0.007). There was no significant difference in survival between HIV-positive and HIV-negative CCPs. Older age (HR = 2.01; 95% CI, 1.01,-4.05), advanced disease (HR = 2.6; 95% CI, 1.67–4.04) and baseline anemia (HR = 1.65; 95% CI, 1.24, 2.20) were independent predictors for higher risk of death.

**Conclusion:**

Survival rates of CCPs did not differ according to HIV status. The risk of death was higher for patients with older age, advanced disease and anemia. HIV patients should be screened for CC according to guidelines to avoid late presentation.

## Background

Cancer and other non-communicable diseases (NCDs) have become leading causes of disability and death in developing countries, including Ethiopia [1]. Cervical Cancer (CC) is a leading cause of cancer morbidity and mortality in women globally. In 2012, 528, 000 new cases and 270,000 deaths were estimated to have occurred worldwide, with the majority of these cases and deaths (90%) occurring in low- and middle-income countries [2]. In Ethiopia, CC is the second most commonly diagnosed cancer and the leading cause of cancer death in women, with about 8000 newly diagnosed cases and 4700 deaths every year [3]. Most CC patients in Ethiopia seek healthcare at an advanced stage, when the effectiveness of treatment is limited [4].

Women infected with HIV are presumed to be more likely to have high risk Human Papilloma Virus (HPV) and have at least a 10% higher risk of developing CC [5–8]. HIV-positive patients are reported to more likely present with advanced stages of CC. It has been shown that HIV changes the natural history of HPV infection, resulting in a rapid progression to invasive lesions, and are associated with adverse survival probabilities [9]. The overall HIV prevalence in adult population of Ethiopia was 1.18% in 2016 with highest prevalence in Addis Ababa and Gambela regions with 4.9 and 4% respectively [10].

However, in the current context, due to the availability of ART and care services, an improved survival and treatment outcome of cervical cancer patients with HIV infection is expected. There is no adequate information documenting this evidence in Ethiopia. Hence, we conducted a retrospective cohort study to assess the survival rate of cervical cancer patients according to HIV status. We reviewed 1655 charts of women with cervical cancer from Tikur Anbessa University Hospital in Addis Ababa, Ethiopia.

## Methods

### Study design and population

We conducted a retrospective cohort study among cervical cancer patients diagnosed at Tikur Anbessa (Black lion) Specialized Hospital (TASH) from September 2008 to September 2012. TASH is the national teaching and referral hospital with more than 800 beds and offers diagnosis and treatment for approximately 400,000 in-patients and out-patients a year. The hospital receives patients who are referred from across the country, as well as patients from Addis Ababa. The hospital is the only one with a radiotherapy facility in Ethiopia. Patients were treated with surgery in the early stages and according to locally adapted guidelines at the radiotherapy center. Brachytherapy was not available at the time. Patients also may have registered but there after not received any treatment. Demographic and clinical characteristics of the patients were retrieved from individual patient charts. The survival status of patients was collected from the cancer registry which obtained information from patient cards or via telephone calls,

### Study variables and data collection

#### HIV status identification

The HIV status of each patient was retrieved from medical charts by trained medical staff. Only half of the patient’s charts contained a registered HIV status. HIV status was documented if the patient had been screened for HIV. Since September 10, 2011 every patient registered at TASH had been screened for HIV on a regular basis. Before this, only patients with a high risk profile (e.g., HIV-positive partner) or clinically suspicious patients were screened. The HIV status was tested using the enzyme-linked immunosorbent assay method. We grouped HIV status into three categories: HIV-positive, HIV-negative and HIV unknown.

### Study outcome and definition

The primary objective of this study was to compare the overall survival of CC patients according to HIV status. We estimated follow-up time between the date of first presentation and the last date of observation. The last date of observation was defined as either death or censoring at the last known alive.

### Data analysis

STATA version IC/14 (StataCorp, College Station, TX, USA) was used for statistical analyses. The overall survival of HIV positive and negative patients was estimated using Kaplan-Meier methods. Kaplan-Meier estimates were compared using the log-rank test. Cox proportional hazards regression model was used to identify predictors for survival. Influences of prognostic factors were estimated using hazard ratios with 95% confidence intervals (CI).

### Ethical considerations

Ethical approval was obtained from the institutional review board of the College of Health Sciences, Addis Ababa University and Martin Luther University, Halle Germany. The confidentiality of the patient status was maintained by avoiding personal identifiers during analysis.

## Results

**Fig. 1:**
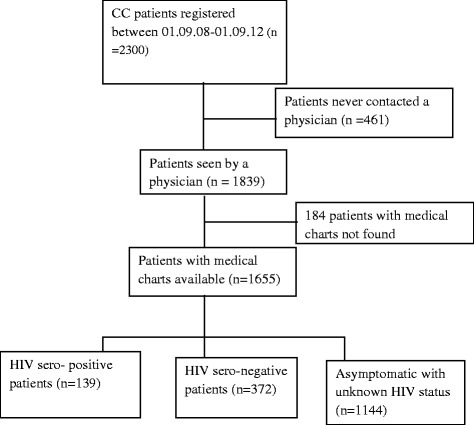
Total number of cervical cancer Patients from radiotherapy center at TASH, Addis Ababa, Ethiopia, included in the final analysis (Strobe diagram)

Of the 1839 cervical cancer patients registered and seen by physicians at TASH, medical charts were retrieved for 1655 (90.0%). Of the 1655 patients with a medical chart, 139 (8.4%) were HIV-positive, 372 (22.5%) were HIV-negative and 1144 (69.1%) were asymptomatic with unknown HIV status (see Fig. 1).

Of all patients, 1081(65.3%) patients received any form of radiotherapy, 190 (11.5%) underwent surgery and 206 (12.4%) received chemotherapy. Among patients treated by radiotherapy, non- radical radiotherapy was provided for 770 (71.0%) of stage IIIB and IVA patients. Of the 190 patients who underwent surgery, 155 (81%) received radical hysterectomy and nine received a simple hysterectomy (see Table 1).

**Table 1:** Demographic and clinical characteristic of cervical cancer patients according to HIV status, TASH, Addis Ababa, Ethiopia, 2008–2012

Patient demographic characteristics	HIV status		
	HIV negative n (%)	HIV positive n (%)	HIV unknown n (%)
Residence			
Urban	128 (34.4)	82 (59)	431 (37.7)
Rural	244 (65.6)	57 (41)	713 (62.3)
Marital status			
Single	3 (0.8)	5 (3.6)	4 (0.4)
Married	297 (79.8)	113 (81.3)	928 (81.1)
Unknown	72 (19.4)	21 (15.1)	212 (18.5)
Age category			
< 30	15 (4.0)	29 (20.9)	47 (4.1)
30–39	81 (21.8)	58 (41.7)	243 (21.2)
40–49	131 (35.2)	40 (28.8)	389 (34)
50–59	88 (23.7)	10 (7.2)	295 (25.8)
60+	57 (15.3)	2 (1.4)	170 (14.9)
FIGO stage at presentation			
I-IIA	59 (15.8)	10 (7.2)	95 (8.3)
IIB-IIIA	148 (39.8)	57 (41.0)	51 (45.1)
IIIB-IVA	144 (38.7)	62 (44.6)	458 (40.0)
IVB	6 (1.6)	5 (3.6)	15 (1.3)
Post-operative	7 (1.9)	2 (1.4)	23 (2.0)
Recurrence	7 (1.9)	3 (2.2)	28 (2.5)
Unknown	1 (0.3)	0	9 (0.8)
Anemia Status			
No anemia ≥12	186 (50)	51 (36.7)	437 (38.2)
> 10 and <12	93 (25)	38 (27.3)	373 (32.6)
8–10	36 (9.7)	24 (17.3)	153 (13.4)
> 5 and <8	24 (6.5)	12 (8.6)	73 (6.4)
< 5	15 (4.0)	12 (8.6)	60 (5.2)
Unknown	18 (4.9)	2 (1.4)	48(4.2)
Co-morbidity status			
No co morbidity	341 (91.7)	124 (89.2)	1045(91.3)
Any co morbidity	31 (8.3)	15 (10.8)	99 (8.7)
Treatment modalities			
Radiation	187 (63.8)	105 (79.5)	789 (75.0)
Surgery	71 (24.2)	12 (9.0)	107 (10.0)
Chemotherapy	35 (12.0)	15 (11.5)	156 (15.0)
Patient outcome			
Alive	331 (89.0)	108 (77.7)	930 (81.3)
Dead	41 (11.0)	31 (22.3)	214 (18.7)
Total	372	139	1144

### Patient characteristics

Table 1 shows the demographic and clinical characteristics of patients according to their HIV status. The mean age of all patients at entry was 49 years. The majority of cervical cancer patients with HIV-positive status came from urban areas (59%), while the majority of patients with HIV-negative, HIV unknown status were rural residents (62 and 66%, respectively). The majority of HIV-positive cervical cancer patients were between 30 and 39 years old (42%), with mean age of 39 (SD = 9) whereas HIV negative, HIV unknown cervical cancer patients were between 40 and 49 years (35 and 34%, with mean age of 50, respectively). About 81% of cervical cancer patients were married. The International Federation of Gynecology and Obstetrics (FIGO) stage at presentation for HIV-positive patients was IIB-IIIA (41%) and IIIB-IVA (44.6%); for HIV-negative/unknown patients IIB-IIIA (40 and 45%, respectively) and IIIB-IVA (39 and 40%, respectively). A total of 120(86.3%) of HIV positive women were on ART.

### Survival according to HIV status

A total of 286 (17.3%) cervical cancer patients died during the follow-up period. The median survival time was 38 months.. Of the total deaths, 41(11%) and 31(22.3%) were HIV negative and HIV Positives. The median survival of HIV positives and HIV negative was 29 and 28 months respectively.

**Fig. 2:**
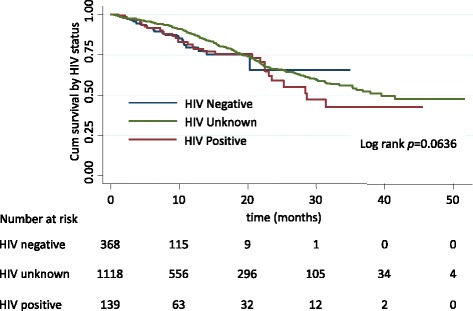
Kaplan-Meier estimates of survival of cervical cancer patients according to HIV status among TASH patients, Addis Ababa, Ethiopia, 2008–2012

Crude survival probabilities did not differ between patients according to HIV status. After adjusting for place of residence, age, FIGO stage, co-morbidity (yes/no) and baseline anemia status, no difference in survival probability was seen between HIV-positive and HIV-negative/unknown cervical cancer patients (HR = 1.16, 95%CI 0.70–1.91) (see Fig. 2).

### Survival according to other clinical and demographic characteristics

After adjusting for place of residence, HIV status, FIGO stage, co-morbidity and baseline anemia status, older CC patients had a two-fold higher risk of death than younger patients (HR = 2.02, 95%CI: 1.01–4.05). CC patients with higher cancer stage (FIGO IIB-IIIA and recurrence) had a higher risk of death, with HR = 2.60 (95%CI: 1.67–4.04) and HR = 2.77 (95%CI: 1.35–5.68), respectively, compared to those with a lower stage. Cervical cancer patients with anemia at baseline were more likely to die than non-anemic patients with HR = 1.65 (95%CI: 1.24–2.20) and HR = 1.84 (95%CI: 1.27–2.66) for a hemoglobin level of greater than 10 and between 8 and 10, respectively. Place of residence and co-morbidity status did not show any differences in overall survival of CC patients (see Table 2).

**Table 2:** Demographic and clinical characteristic associated with the survival of cervical cancer patients, TASH, Addis Ababa, Ethiopia, 2008–2012

Characteristics	Unadjusted HR (95% CI)	Adjusted HR (95% CI)	*P*-value
HIV status			
Positive	1.00	1.00	
Negative	1.13(0.70,1.81)	1.16(0.70,1.91)	0.564
Unknown	0.77(0.53,1.13)	0.76(0.51,1.15)	0.206
Residence			
Rural	1.2(0.94,1.51)	1.16(0.91,1.48)	0.212
Urban	1.00	1.00	
Age group			
< 30	1.00	1.00	
30–39	1.52(0.78,2.97)	1.43(0.73,2.82)	0.290
40–49	1.50(0.78,2.86)	1.47(0.75,2.87)	0.259
50–59	1.12(0.57,2.20)	1.17(0.58,2.36)	0.650
60+	1.83(0.93,3.61)	2.01(1.01,4.05)^a^	**0.049**
FIGO stage at presentation			
I-IIA	1.00	1.00	
IIB-IIIA	1.32(0.86,2.03)	1.22(0.79,1.88)	0.372
IIIB-IVA	3.07(2.00,4.71)	2.60(1.67,4.04)^a^	**<0.001**
IVB	3.77(1.13,12.55)	2.54(0.74,8.68)	0.137
Post-operative	1.17(0.45,3.07)	1.04(0.40,2.74)	0.930
Recurrence	2.46(1.21,5.00)	2.77(1.35,5.68)^a^	**0.005**
Anemia: Hgb level at presentation (g/dl)			
No anemia ≥12	1.00	1.00	
> 10 and <12	1.82(1.38,2.40)	1.65(1.24,2.20)^a^	**0.001**
8–10	2.20(1.54,3.14)	1.84(1.27,2.66)^a^	**0.001**
< 8 and ≥5	1.47(0.90,2.42)	1.35(0.81,2.25)	0.242
< 5	1.24(0.67,2.31)	1.12(0.60,2.10)	0.706
Unknown	1.74(0.23,12.73)	1.21(0.16,8.96)	0.846
Co-morbidity status			
No co-morbidity	1.00	1.00	
Any co-morbidity	0.98(0.68,1.40)	0.99(0.68,1.43)	0.969

*HR* hazard ratio, *CI* confidence interval, *HIV* Human Immune deficiency Virus, *FIGO* International Federation of Gynecology and Obstetrics

values in boldface are statistically significant at alpha of 0.05

## Discussion

This study showed that CC patients with known HIV infection constituted 8.4% of all CC patients registered in the largest referral hospital in Ethiopia. The survival rate was similar among CC patients with and without known HIV infection in this cohort where only 14% of patients received any form of therapy considering only the curative radiotherapy. The majority of patients with known HIV infection came from the urban area, compared to patients without HIV infection. About 86% of the HIV positive CCPs were on ART. A slightly higher proportion of patients with HIV infection presented with late-stage cancer. The median time of observation in patients without event was 38 months. Older age, late-stage disease and anemia were factors significantly influencing overall mortality probabilities of cervical cancer patients.

The survival of CC patients with positive HIV status was similar to those with negative or asymptomatic with unknown HIV status. The observed survival of HIV positive patients could have been compromised for two reasons: either because they die due to the HIV infection or second because the CC is more aggressive. Since we do not have information on cause of death in our study, we can only suggest that the widespread use of ART in this cohort may prevent HIV related deaths and also fast progression of CC [11–13].

A higher proportion of HIV-infected cervical cancer patients presented with advanced stages of cancer compared to those with negative/unknown HIV status. This is probably because the HIV infection decreases the progression time of cervical cancer to more advanced stages [9, 14].

In our Cox model, we found older age, baseline anemia and advanced stage to be significantly associated with higher all-cause mortality of cervical cancer patients. This finding is consistent with studies conducted in similar settings elsewhere. According to a Nigerian study, there was a 41% higher proportion of death for advanced stages compared to early stages [15]. Moreover, a Kenyan finding showed that the 2-year survival of cervical cancer patients at advanced stage was less than 20% [16].

Baseline anemia independently predicted a higher risk of death; moderate anemia was significantly associated with higher mortality compared to patients with no anemia. In this study, anemia was defined as a hemoglobin level below 12 g/dl. Another similar study from north-central Nigeria indicated baseline anemia to be an independent predictor of lower survival in cervical cancer patients [15]. Furthermore, older (≥60 years) patients had a significantly higher risk of death compared to younger patients. Several other studies have also reported this [17, 18]; this might be due to fact that young patients are more likely to respond to treatment and present in an early stage [17]. In addition, it has to be considered that the probability for all-cause death is higher in the older age groups.

The strength of this study is the large number of all CC patients with medical charts available during a 4-year period in TASH, the only hospital for cancer treatment in Ethiopia and the inclusion of those patients who only registered but never received treatment were included. However, a limitation is the large proportion of CC patients with only asymptomatic with unknown HIV status. Since their characteristics were very similar to those with negative HIV sero-status we assume they are more likely to be HIV sero-negative. However, this may have underestimated the effect of HIV on the survival of CC patients. Moreover, about 20% of all cervical cancer patients who registered at TASH and were scheduled to see a physician for treatment planning during the study period did not come back; these patients may have had very advanced disease. We assume that the 10% of charts missing were random cases due to problems of misplacing, or miss-spelling names or numbers. The huge efforts that would have been needed to retrieve these charts were out of scale.

## Conclusion

In conclusion, known HIV-positive patients constitute a considerable proportion of CC patients in a hospital cohort in Ethiopia and are diagnosed at a more advanced stage of disease compared to those with negative and unknown status. Survival did not differ between HIV-positive and HIV-negative and -unknown CC patients after adjusting for other prognostic factors. The high proportion of advanced stage cancer in HIV-positive patients suggests the need to increase the implementation and awareness of cervical cancer screening among HIV-positive women and remove barriers to accessing screening. Anemia at presentation probably reflects the severity of disease and therefore shows adverse survival. Attention should be given generally to those CC patients who are diagnosed at older age and with advanced stage of disease, as these are contributing factors for lower survival rates. Finally, a high proportion of unknown HIV status justifies screening for HIV in all CC patients, this is now in place.

## Abbreviations

ART: Antiretroviral Therapy
CC: Cervical Cancer
CCPs: Cervical Cancer Patients
CI: Confidence Intervals
FIGO: International Federation of Gynecology and Obstetrics
HIV: Human Immune Deficiency Virus
HPV: Human Papilloma Virus
HR: Hazard Ratio
NCDs: Non-communicable Diseases
SD: Standard Devation
TASH: Tikur Anbessa Specialized Hospital
